# Oral Feeding of Probiotic* Bifidobacterium infantis*: Colonic Morphological Changes in Rat Model of TNBS-Induced Colitis

**DOI:** 10.1155/2016/9572596

**Published:** 2016-04-03

**Authors:** Najma H. Javed, Musaad B. Alsahly, Jagdish Khubchandani

**Affiliations:** ^1^Department of Biology, Ball State University, Muncie, IN 47306, USA; ^2^Department of Physiology and Health Science, Ball State University, Muncie, IN 47306, USA

## Abstract

Ulcerative colitis (UC) is a chronic inflammatory bowel disease of unknown etiology. It has been proposed that modifying the bacterial flora in intestine with probiotics may decrease the inflammatory process and prevent relapses in UC. We investigated the possible protective and therapeutic effects of a single strand of probiotic,* Bifidobacterium infantis* (BI), on colonic inflammation, in rats with regular feedings. Two groups of Lewis rats were prepared (*n* = 8). The first group was the control, sham-fed group (*n* = 4). The other group was the experimental BI-fed group (*n* = 4). Colitis was induced in both groups by intrarectal administration of TNBS under light anesthesia. The sham-fed colitis induced groups received a daily oral gavage feeding of 1.0 mL distilled water, whereas the* B. infantis*-fed group received 0.205 g of* B. infantis* dissolved in 1.0 mL distilled water daily. The change in body weight and food and water intake was recorded over the course of each study and analyzed. The rats were euthanized and tissues from the descending colon were harvested and analyzed microscopically and histologically. Results of our study indicated significant reduction in inflammation, mucosal damage, and preservation of goblet cells, as compared to the control animals. Modulation of gastrointestinal (GI) flora suggests a promising field in developing strategies for prevention and treatment of inflammatory bowel diseases by dietary modifications.

## 1. Introduction

The stomach contains few bacteria because of its high acidity and protective secretions of the upper GI, but certain strains of bacteria travel through the GI tract and settle in the intestines, specifically, the colon [1, 2]. Approximately, twenty percent of the feces of a normal human consists of bacteria, most of which have come from the colon. While it takes about 3-4 hours for food to travel through the small intestine, it takes 24–48 hours for food to travel through the colon. This slower flow rate provides bacteria in the colon with time to reproduce so that they gain high concentration. In the colon of normal people, bacteria make up most of the flora and more than 60% of the dry mass of feces. The main bacteria in the colon are* Lactobacillus*,* Streptococcus*,* Bifidobacterium*,* Eubacterium*,* Coliforms*,* Bacteroides*, and* Clostridium*. A healthy person carries about 100 trillion microorganisms in the intestines [1, 2].

Typically, these colonic microbes are harmless and often times are beneficial. The microbial flora is important for maturation of the immune system by its ability to outcompete pathogenic microbes for nutrient and space. Also, the microbial flora plays a role in development of normal colonic morphology and maintenance of a sustained inflammatory response [1, 2]. Furthermore, the microflora also supports the barrier function of the colonic mucosa, preventing the entry of pathogenic microorganism and allergens [1–4].

## 2. Inflammatory Bowel Diseases (IBD)

When the GI tract is inflamed, there is a collapse of intestinal barrier function, irregular secretions, and changes in the motility patterns which participate in symptom generation [5]. Typically, changes in GI function that accompany GI inflammation give rise to diarrhea, cramping, and pain which are standard symptoms of inflammatory bowel disease (IBD) [6]. IBD which consists of two major types, ulcerative colitis (UC) and Crohn's disease (CD), affects almost 3.5 million people in the United States and Europe. Ulcerative colitis is a chronic inflammatory disease that causes inflammation and ulcers in the top layer coating the large intestine. Typically, people with this condition develop small abscesses that flare up periodically causing abdominal tenderness, loss of appetite, weight loss, diarrhea, abnormal bowel habits, and bloody stool [7, 8]. Although extensive progress has been made, a major gap in awareness of the pathogenesis of IBD still remains. Without further studies on the pathogenesis of IBD, the finding of lasting and effective strategies of treatment is impossible [7, 8].

Recent studies propose that IBD has a multifactorial etiology that includes adjustments in GI motility, small-bowel bacterial overgrowth, microscopic inflammation, and visceral hypersensitivity. Some of these suggested components of IBD pathophysiology may possibly lend themselves to probiotic therapeutic benefits [7–9].

## 3. Probiotic Therapy in GI Disorders

Many GI diseases can occur due to imbalance in the gastrointestinal flora. Probiotic therapy is the practice of administrating beneficial live microorganisms in adequate amounts as a dietary supplement. This can either change the composition or metabolic activities of the microbiota or modulate host immune system reactivity in order to receive the proposed health benefits [7–10]. The microorganisms used in probiotic therapy are often bacteria. Lactobacillus acidophilus and Bifidobacterium bifidum are the most common probiotic bacteria. These microorganisms are very similar to beneficial bacteria that assist in digestion process and are found naturally in the GIT [11]. Although the accurate mechanism of how probiotics may help in the reduction of symptoms in IBD is unknown, the effects of probiotics on alterations in GI bacteria appear to play a part. The properties of different probiotic species differ and the effects of specific probiotic strain should not be generalized to others without confirmation in isolated studies [7, 8].

## 4. *Bifidobacterium infantis*

*Bifidobacterium infantis* is a member of the* Bifidobacteria* family, a strain of bacteria that is normally found in the human intestines. Because these bacteria do not normally cause infections, they can be used in probiotic supplements. Recent studies indicate that* B. infantis* may also be effective in treating irritable bowel syndrome (IBS) [8–11].* Bifidobacterium infantis* alters the balance of several types of bacteria in the GI tract in ways that may help reduce inflammation. Some early clinical studies reported the valuable effects of these bacteria on the symptoms of irritable bowel syndrome (IBS) including flatulence, diarrhea, constipation, urgency, and visceral pain.

The* Bifidobacteria* are thought to have actual physiological effects on GI tract. First, the most significant postulated physiological effect is the action of gut conditioning. The gut conditioning action is the general effect of improving the gut environment, which includes improvement of the gut flora, suppression of gut putrefactive substances, and improvement of the fecal properties and defecation state (remission of constipation or diarrhea) [8, 11, 12]. Also,* Bifidobacteria* produce acetic acid with a strong bactericidal action, which impedes the growth or colonization of harmful bacteria within the colon. Another important effect of* Bifidobacteria* is the immune system modulation.* Bifidobacterium infantis* can beneficially modulate immune system of the host, but the exact mechanism is still unknown. The specific effects of BI on colonic morphology have not yet been studied [8, 11, 12]. Therefore, additional investigation of BI and its relationship with IBD is required.

## 5. Objective

Since the last decade, research on probiotics has expanded tremendously. Researchers have tried to explore ideal types of probiotic combinations that would be beneficial for patients suffering from various gastrointestinal disorders. The main objective of this study was to test the hypothesized medical benefits of probiotic BI on IBD in a rat model. In this research project, we tested the hypothesis that the oral feeding of* Bifidobacterium infantis* has an effective role in reducing the inflammation of ulcerative colitis.

Functional studies in have demonstrated that probiotics can decrease inflammation in the GI tract. However, the exact mechanism through which this occurs is still unclear. Many studies have not dealt with clear correlation of functional aspects of the probiotics with the morphology of the inflamed colon and structural integrity. Also, the properties of any two probiotics are not the same and therefore, cannot be generalized to others without confirmation in separate studies. Therefore, we focused on a single strand of probiotic,* Bifidobacterium infantis* (BI), that we have used previously for functional studies and correlated it with the structural and morphological changes in animal model of TNBS colitis.

## 6. Methods and Materials

### 6.1. Experimental Design

Eight Albino male Lewis rats of similar age (8-9 months) and weight (350–450 grams) were obtained. All rats were randomly divided into two populations (*n* = 4 each). The rats were tail-marked for identification and placed into the sham-fed colitis induced group or the probiotic-fed (*Bifidobacterium infantis*) colitis induced group. All rats were maintained in standard conditions with free access to food and water that were refreshed every day. Animal protocol was approved by the institutional Animal Care and Use Committee at Ball State University, USA.

Ten days prior to colitis induction, probiotic was added to the* B. infantis*-fed colitis group's water supply. Both groups of animals were monitored daily and weight, water/food intake, and appearance were documented. On day three, seven days prior to the colitis induction, the rats were fed orally one time per day through curved oral gavage. After ten days (3 days water supply and 7 days oral gavage), the rats were induced with ulcerative colitis using TNBS that lasted for seven days with continued oral feedings. Finally, at the conclusion of seven days, all the rats were euthanized and tissues from the descending colon were harvested for histological analysis.

Trinitrobenzene sulfonic acid rat model is one of the most widely used models to study the pathogenesis of IBD [8, 13]. Induction of colitis in animals can be achieved by intrarectal administration of a chemical agent called 2,4,6-trinitrobenzenesulfonic acid (TNBS) into the rat colon. This agent induces inflammation and several alterations in the colon morphology with features similar to those found in chronic inflammatory diseases in humans [13, 14].

### 6.2. Experimental Protocol

#### 6.2.1. Oral Feedings

The sham-fed colitis induced group received daily feeding by oral gavage of 1.0 mL distilled water, while the* B. infantis-*fed group received 0.205 g of* B. infantis* dissolved in 1.0 mL distilled water every day. BabyLife, by Solaray, is a* B. infantis* powder that has 3 × 10^9^ colony forming units (CFU) of* Bifidobacterium infantis* per 615 mg of powder. Oral feedings required a 1 × 10^9^ CFU (54) dose, which contained 0.205 g of BabyLife* B. infantis* in 1 mL of water for the feedings. Feeding was done while animals were induced with light anesthesia with IsoVet (Isoflurane, USP) (Schering-Plough Animal Health Corp.). The loose skin of the back of the rat was grasped and the rat's tail was restrained with ring finger and little finger. Then, the feeding gavage tube was introduced from the pharynx into the esophagus when the rat is in the act of swallowing. When desired length of insertion was achieved, the material was injected [8]. After the procedure, each rat was observed for signs of distress, such as gasping or frothing of the mouth.

#### 6.2.2. Induction of Colitis

Seven days after oral feedings, the rats were fasted overnight but had free access to water. Briefly, the rats were lightly anesthetized with IsoVet (Isoflurane, USP) (Schering-Plough Animal Health Corp.) and given an enema of 1.0 mL 5% (w/v) 2,4,6-trinitrobenzene sulfonic acid (TNBS) (Sigma-Aldrich). TNBS was administrated into the lumen of the colon through a polyethylene rubber catheter inserted rectally 8 cm proximal to the anus. TNBS administration was followed by 1.0 mL of air and the rats were then kept in the Trendelenburg position for five minutes to ensure acute colitis induction [8]. The colitis conditions and features lasted seven days. Excess discomfort was continually assessed and data were collected and documented for weight loss, blood loss, and stool activity so that it could be evaluated on a Disease Activity Index (DAI) [8].

#### 6.2.3. Tissue Harvesting and Macroscopic Assessment

After seven days of UC induction, all rats were euthanized with an overdose of IsoVet, followed by a thoracotomy. The whole distal colon of each rat was surgically removed and gently cleaned of fat and mesentery, snap frozen in liquid nitrogen, and stored in −90°C until it was used for slicing and histological examination at a later time. Every tissue was assessed by two researchers for macroscopic features prior to freezing. The criteria focused on vascularity and appearance, adhesions, diarrhea, and presence of megacolon if any.

#### 6.2.4. Colonic Tissue Slicing

A cryostat (MICROM HM505N) was used to slice 15 *μ*m thick sections. The cryostat was cleaned prior to use. Small plastic molds were used individually for each sample. A small amount of OCT (mounting media for frozen sections) was filled in the mold. A quarter inch of a tubular piece of colon tissue was placed upright and was covered by OCT in the mold, fixed, and frozen. The tissue was allowed to completely freeze at −23°C. The sample was then mounted on slicing stage of the cryotome and sliced into 15 *μ*m thick sections. A paintbrush was used to pull down the desired section of the tissue. The desired slice was then picked up by placing a polysine microscope slide (Thermo Scientific) facedown onto the sectioned tissue. Three to four serial sections were placed on each glass slide. Slides were kept at −23°C and allowed to equilibrate to room temperature prior to hematoxylin and eosinophil (H&E) stain.

#### 6.2.5. Assessment of Colonic Damage

The severity of colitis was evaluated in three ways: macroscopic damage scoring, histological damage, and staining with H&E. In addition, the change in body weight, food and water intake was recorded over the course of each study. The criteria for scoring of macroscopic damage have previously been described by Stucchi et al. (Table 1) [15]. Every tissue was assessed by two researchers in a blinded fashion and scored through macroscopic scoring criteria for scoring UC severity. The criteria for assessment of colonic damage focused on ulceration size and appearance, adhesions, and diarrhea. On the other hand, histological scoring was based on a quantitative scoring system in which the following features were considered and scored as follows: extent of destruction of normal mucosal architecture (0, normal; 3, maximal damage), presence and degree of cellular infiltration (0, normal; 3, maximal infiltration), extent of muscle thickening (0, normal; 3, maximal thickness), presence or absence of crypt abscesses (0, absent; 1, present), and the presence or absence of mucus depleted goblet cells (0, absent; 1, present). In each case, a numerical score was given with a maximum score of 11 for all criteria [16].

### 6.3. Statistical Analysis

All histological assessments were performed using coded slides to prevent observer bias. Data was expressed as means ± SEM. Independent sample *t*-tests were used to test for the significance between two group means of the morphological tissue response. Results were considered significant at *p* ≤ 0.05. Observational results were compared using an ANOVA (with *n* = 4/group = 8) to evaluate the BI-fed and sham-fed rats, before and after the induction of colitis data.

## 7. Results

TNBS colitis induction was successful in showing experimental colitis in all eight Lewis rats. 10~24 h after administration of TNBS, rats in both groups began to show such symptoms as obvious diarrhea, body weight loss, inactivity, and anorexia. Oral BI and sham feedings were also successful throughout the feeding period. The hunchback appearance of the majority of animals in the sham-fed group for 48 h after TNBS-induction was an indication of severe abdominal pain. Disease Activity Index (DAI) began to decrease in the BI feeding group on the second day. BI feeding was effective in alleviating the symptoms of diarrhea in TNBS-induced colitis in rats. The rats successfully provided observational, macroscopic, and histological data for analysis.

### 7.1. Observational Data: Weight, Food Intake, Water Intake, and Disease Activity Index

**Figure 1:**
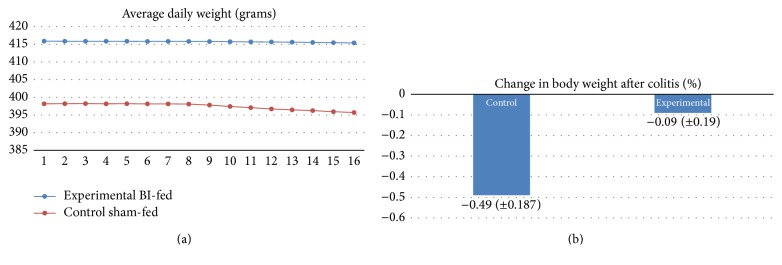
(a) Average daily weight. Even before TNBS induction, the weight of rats from both groups declined. Weight loss between the groups was statistically insignificant prior to TNBS induction. A sharp decrease in weight is seen between Days 8 and 9 because of the overnight fasting. Data expressed as daily group means ± SEM. (b) Change in % body weight from Day 1 to Day 7 time course of ulcerative colitis induction. Data expressed as means ± SEM. The percent weight loss was calculated from Day one to Day 7 of colitis. The BI-fed rats lost significantly less weight over the ulcerative colitis time period. Tested with an independent sample *t*-test (*n* = 4/group) (*p* < 0.05).

**Figure 2:**
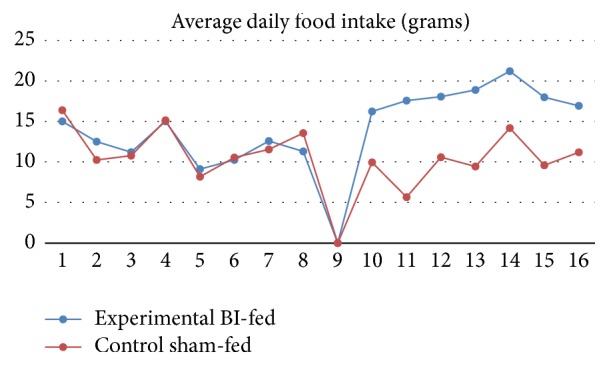
Average daily food intake. Food intake prior to TNBS induction was not significantly different. After the first six days of ulcerative colitis, the BI-fed rats ate significantly more food (^*∗*^ *p* < 0.05).

**Figure 3:**
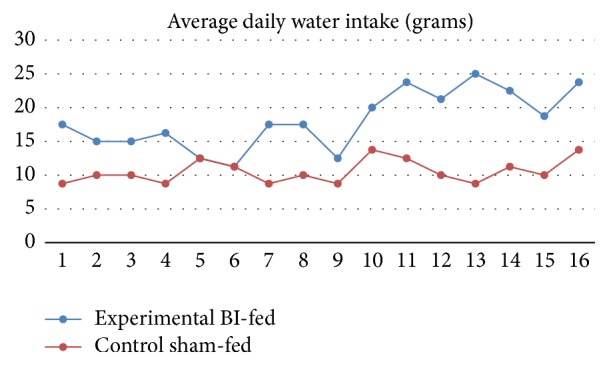
Daily average water intake. Water intake was significantly higher in BI-fed than the sham-fed rats before and after TNBS induction (^*∗*^ *p* < 0.001). BI-fed rats drank significantly more water after TNBS than before TNBS (*p* < 0.001).

Before TNBS induction, body weight change was statistically insignificant between the BI-fed and sham-fed rats (*p* = 0.3), but after the TNBS administration to induce colitis, the BI-fed rats presented with a significantly decreased percent body weight as compared to the sham-fed rats group (*p* < 0.05) (Figures 1(a) and 1(b)). The weights correlated with the food consumption, because our data showed that after the first five days of colitis induction the BI-fed rats ate significantly more food than the sham-fed rats (*p* < 0.05) (Figure 2). These results propose a reduction of UC symptom with BI feedings. Also, this trend can be seen on the Disease Activity Index scores (DAI) during the prefeedings and TNBS period. The observational records showed that water intake for the BI-fed rats was significantly more before and after TNBS (*p* < 0.001) when compared to the sham-fed rats (Figure 3). Strangely, the data showed that BI-fed rats drank significantly more water after TNBS than before (*p* < 0.001), but the sham-fed rats showed no significant difference between water consumption before and after TNBS induction (*p* = 0.095). Statistically, observational results were compared using an ANOVA (*n* = 4/group) to assess the BI-fed and sham-fed rats, before and after TNBS data.

### 7.2. Macroscopic Scoring of Colonic Inflammation and Examination of Other Organs

**Figure 4:**
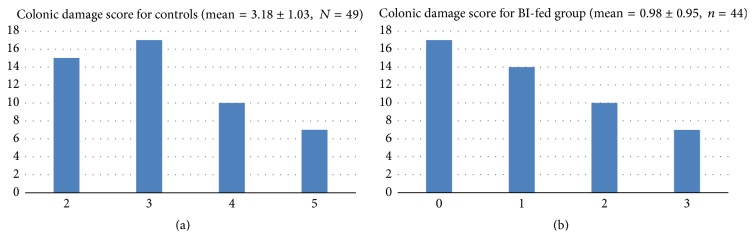
(a) Colonic damage score for controls. The colonic damage was evaluated for all parameters in sham-fed rats. The microscopic damage score of sham-fed animals was elevated compared to BI-fed rats (tested with an independent sample *t*-test). (b) Colonic damage score for BI Group. The colonic damage was evaluated in all parameters in BI-fed group. The microscopic score of BI-fed animals was lesser than sham-fed animals (tested with an independent sample *t*-test).

Visual observation scoring of colon tissues in both different groups showed significant morphological inflammatory changes in the BI-fed versus sham-fed colitis rats (independent sample *t*-tests weighted, *p* = 0.001) (Figures 4(a) and 4(b)). Obvious hyperemia, edema, swelling, and ulceration could be seen on the colonic mucosal surface of the sham-fed rats. In contrast, these lesions were greatly relieved in the BI-fed rats.

Results obtained from the macroscopic colonic inflammation propose that BI decreases inflammation, which is evident from the reduction in the colonic ulceration, absence of colonic and peritoneal adhesions, and wall thickness in the BI-fed rats (Table 2).

### 7.3. Histological Scoring of Colonic Inflammation

**Figure 5:**
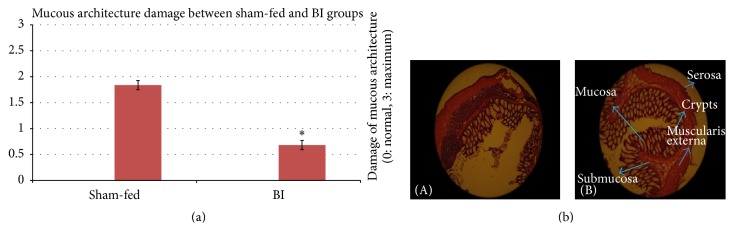
(a) The mucosal architecture damage between sham-fed group and BI group. The mucous architecture damage was evaluated between the sham-fed and BI-fed groups. The BI-fed rats showed significantly less mucous damage (tested with an independent sample *t*-test) (^*∗*^ *p* < 0.05). (b) The mucosal architecture damage between sham-fed group and BI group. (A) The mucous architecture distortion. Sham-fed group (B) showing well-preserved mucous architecture. BI-fed group. H&E ×150.

**Figure 6:**
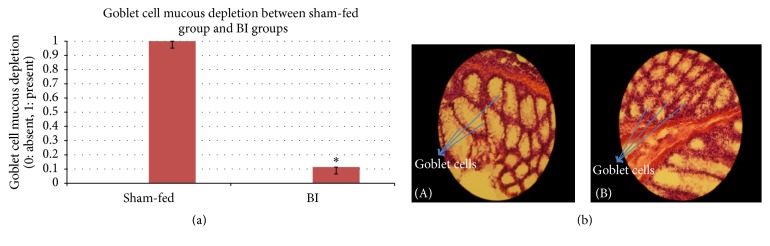
(a) The mucous-depleted goblet cell in sham-fed group and BI group. The goblet cell depletion was evaluated between the sham-fed and BI-fed groups. The BI-fed rats showed significant preservation of goblet cells. Tested with an independent sample *t*-test (^*∗*^ *p* < 0.05). (b) The goblet cell depletion between sham-fed group and BI group. (A) The depletion of goblet cells. Sham-fed group (B) showing well-preserved goblet cells. BI-fed group. H&E ×200.

**Figure 7:**
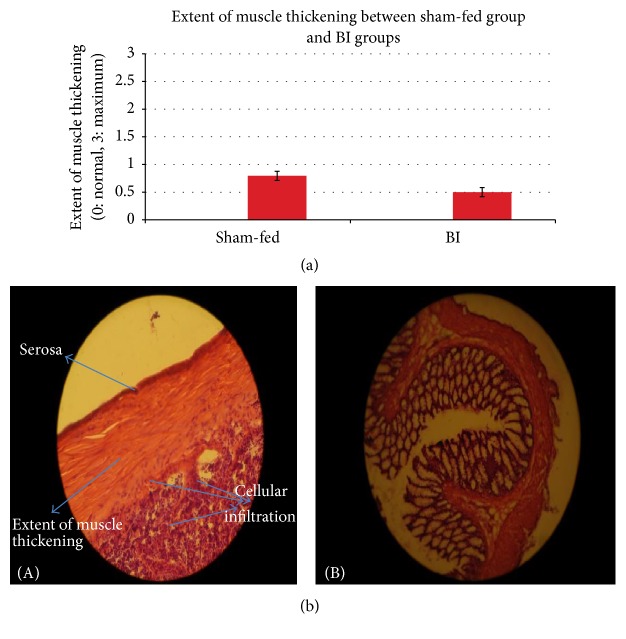
(a) The extent of muscle thickening between sham-fed group and BI group. The extent of muscle thickening was evaluated between the sham-fed and BI-fed groups. Extent of muscle thickening between the groups was not statistically significantly different. However, there was a huge extent of muscle thickening in some sham-fed samples. Tested with an independent sample *t*-test. (b) The extent of muscle thickening between sham-fed group and BI group. (A) The extent of muscle thickening. Sham-fed group (B) showing lesser extent of muscle thickening. BI-fed group. H&E ×200.

**Figure 8:**
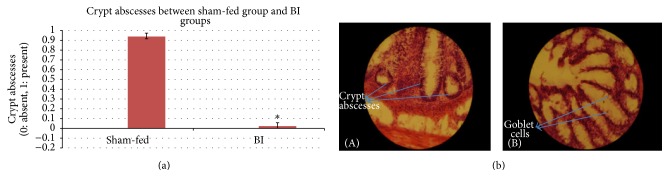
(a) The crypt abscesses between sham-fed group and BI group. The crypt abscesses was evaluated between the sham-fed and BI-fed groups. The BI-fed rats showed significantly less crypt abscesses. Tested with an independent sample *t*-test (^*∗*^ *p* < 0.05). (b) The crypt abscesses between sham-fed group and BI group. (A) Number of crypt abscesses. Sham-fed group (B) showing nearly absent crypt abscesses. BI-fed group. H&E ×200.

**Figure 9:**
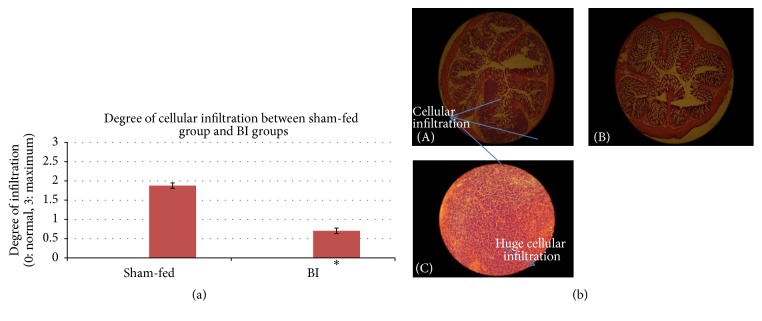
(a) The degree of cellular infiltration between sham-fed group and BI group. The degree of cellular infiltration was evaluated between the sham-fed and BI-fed groups. The BI-fed rats showed significantly less degree of cellular infiltration. Tested with an independent sample *t*-test (^*∗*^ *p* < 0.05). (b) The degree of cellular infiltration between sham-fed group and BI group. (A) The presence of cellular infiltration. Sham-fed group (B) showing almost absent cellular infiltration. BI-fed group. H&E ×150. (C) The cellular infiltration (bigger magnification). Sham-fed group. H&E ×400.

Difference between control and BI group was significant on the most parameters under study. Control group had higher damage compared to BI group. In BI-fed rats, there was significant preservation of mucosal architecture (*p* < 0.001) (Figures 5(a) and 5(b)). There was significant preservation in mucus depleted goblet cells as compared to nonfed control animals (*p* < 0.001) (Figures 6(a) and 6(b)). Some thickening of the muscularis externa accompanied by a slight submucosal edema was also recognized (*p* = 0.013) but no significant difference could be assessed (Figures 7(a) and 7(b)). Histological data showed that presence of crypt abscess was attenuated in the BI-fed animals (*p* < 0.001) (Figures 8(a) and 8(b)). Most of the samples of the BI-fed rats showed almost complete preservation of the epithelial cell layer, in contrast to the extensive ulceration observed in the sham-fed rats. Finally, in BI-fed rates only slight infiltration in the lamina propria, muscularis mucosa, and submucosa was observed (Figures 9(a) and 9(b); *p* < 0.001).

**Figure 10:**
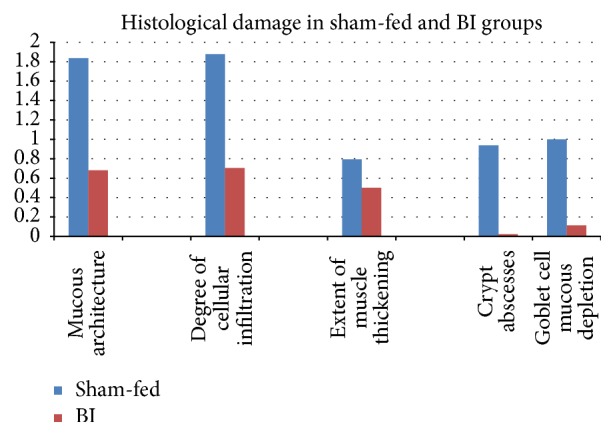
Comparison of the histological damage between sham-fed group and BI-fed group. The histological damage was evaluated in all parameters between the sham-fed and BI-fed groups. The BI-fed rats showed significantly less colon damage. Tested with an independent sample *t*-test (*n* = 4) (*p* < 0.05).

The histological appearance of the colon in the sham-fed rats showed significant damage of the mucosal architecture with obvious reduction in mucous depleted goblet cells. There was evidence of clear mononuclear cell infiltration in the lamina propria, muscularis mucosa, and submucosa. Thickening of the muscularis externa and submucosal edema was also seen. However, muscularis externa thickening could not be clearly differentiated from the BI-fed group. In addition, crypt abscesses were clearly seen in the sham-fed animals (Table 3). The histopathologic score (HPS) of sham-fed animals was pronouncedly elevated (Figure 10) (Table 3).

## 8. Discussion

The ideal therapeutic approach for IBD is still under investigation. However, the concept of probiotics has attracted increasing attention in recent years. A number of studies confirm anti-inflammatory effects of probiotics in TNBS models or patients of IBD [2, 8, 13, 14]. With the increasing attention, there is a requirement for well-documented sources and analysis of probiotic properties. Earlier research has acknowledged the abilities specific probiotic strains have in enhancing the epithelial cell barrier in the gastrointestinal tract and decreasing the inflammation induced by ulcerative colitis. However, no study to date has explained the exact mechanism for how these probiotics work. In a previous study from our laboratory, BI was found to increase secretomotor activity of the ENS, possibly providing protection and prevention of harm by expelling the TNBS chemical before it damages the colon [8]. It was proposed that BI may shield the damaged mucosa caused by inflammation, by increasing secretions which could maintain the integrity of the protective system. However, the specific morphological effects of BI on inflamed colon have not yet been studied and correlated with the secretomotor activity concurrently.

The results obtained from this study found a loss of appetite by significant decrease in food intake and weight loss, supporting trends from earlier studies that reflect the severity of ulcerative colitis [8, 17]. Previous studies from our laboratory truly describe the results of colitis and offer a point of comparison with a nonfed, noncolitis Lewis rat group [8, 17].

Connections with the Disease Activity Index score and macroscopic tissue damage score caused by UC demonstrate a trend for decreased inflammation and symptoms within our BI-fed rats. This uniformity with previous studies shows the usefulness of the TNBS animal model as helpful instrument for advancing our understanding of IBD [18].

The results obtained in the present study are supportive of the helpfulness of the dietary incorporation of probiotics in IBD therapy. Furthermore, this study confirms the GI anti-inflammatory activity which was previously shown by this strain of* Bifidobacterium* [8, 11, 12].

This study reveals the efficacy of probiotic therapy with BI strain in intestinal inflammation by incorporating a new microorganism to the probiotics that have been reported to attenuate the development of colonic injury in experimental and human IBD [11, 12, 19]. Therefore, oral administration of probiotic BI can facilitate recovery from TNBS-induced colonic damage, as it was proved histologically, with a significant reduction in the extent and severity of inflammation in the tissue. This beneficial effect has also been determined biochemically by a reduction in colonic MPO activity, a marker of neutrophil infiltration that has been previously described to be upregulated in experimental colitis and is widely used to detect and follow colonic inflammatory processes [20].

As confirmed through macroscopic scores and histological data, the results of the present study demonstrate that treatment with oral BI markedly reduced the severity of inflammation in TNBS-induced colitis. Furthermore, all parameters showed the presence of inflammation and oxidative injury in inflamed colonic tissue, while BI probiotic feeding indicated a potent anti- inflammatory and antioxidant effect.

There was general colonic mucosal and submucosal damage characterized by infiltration of inflammatory cells and ulcers in sham-fed animals. However, the inhibitory role of BI on the infiltration of inflammatory cells into the colonic mucosa might account for the beneficial effect of this probiotic against tissue injury, because margination and extravasation of circulating granulocytes contribute to the colonic injury in TNBS model. These findings are in agreement with previous studies that described the attenuation exerted by several probiotics in leukocyte-endothelial cell adhesion in this experimental model of rat colitis [8, 11, 12, 21].

Our histological analysis showed well-preserved goblet cells and thus mucous secretion in BI group providing protection and prevention to harm by expelling the TNBS chemical before it damages the colon. That also was evidenced by the clearly intact mucous architecture in the BI group. Since the goblet cells are responsible for the secretion, these results prove the suggestion from previous studies in our lab which indicate that BI may help shield the damaged mucosa by increasing secretions [8]. Furthermore, goblet cell depletion and lack of mucous production in active TNBS could describe the defective mucus barrier in UC, resulting in a collapse of the physical barrier, which allows the luminal microbes to invade the mucosa and trigger inflammation [8, 11, 12, 22]. Also, Caballero-Franco et al. (2007) found that treatment with probiotic bacteria triggered an increase of 60% of basal luminal mucin contents by upregulation of* MUC2* gene expression. In the same study, an increase in number of goblet cells was detected as an effect of probiotic therapy [23].

Significantly higher water intake in BI-fed rats after TNBS induction may suggest a further increase in luminal fluid in an attempt to provide “healthy” flushing of the tissue [8]. The histological results in this study did not indicate any measurable difference in thickening of the colonic muscularis externa in both nonfed and BI-fed animals. Since this is an acute model of colitis, it may take longer for muscle hypertrophy in any of these groups. Researchers have shown a change in entire thickness of the mucosal wall due to edema, accumulation of fat, and hypertrophy of the muscle layer that may have contributed to thickened mucosal wall [24]. This infiltration of inflammatory cells thickens the colon lining and may interfere with absorption and motility or the ability of the colon to contract and move the food. If feeding of BI has an ability to preserve the colon wall thickening, this may be due to decrease in edema and cellular infiltration in the TNBS-BI-fed group [25].

Clusters of neutrophils within a crypt are referred to as crypt abscesses and are frequently connected with crypt destruction. Crypt abscesses were observed in most of the sham-fed group animals providing a typical histological finding of ulcerative colitis which supports the trends of TNBS as an excellent animal model of UC [26]. Over time, chronic inflammation damages the crypts, and the normal architecture of the mucosa is lost, replaced by ulcers and scarring, which can shorten or narrow the colon. In our study, the BI-fed animals did not show any significant presence of abscess formation. This further suggests the reduction in severity of inflammation in response to BI feeding. In our study the strongest finding was the preservation of goblet cells while the weakest finding was the prevention of the extent of muscle thickening alone. These results suggest that BI plays an important role in colonic maintenance process [8].

The results of this study should be considered in light of potential limitations. First, colon slicing and taking sections from the tissue for histological analysis are a sophisticated procedure. We used a paintbrush to pull down the desired section of the tissue. This may have led to aberrant findings. Second, evaluation of the colitis tissue was more difficult than anticipated.

Therefore, tissue was taken directly above the visible ulcerations and adhesions, making it difficult to know if the less damaged tissue was behaving in the exact same way. Finally, the sample size for rats should have been larger to further strengthen our findings.

In conclusion, administration of the probiotic BI facilitates the recovery of the inflamed tissue in the TNBS model of rat colitis. Furthermore, BI supports the maintenance of colonic integrity against chronic inflammatory processes. This study presented a significant role of probiotic* Bifidobacterium infantis* in preserving all colonic morphological components in the animal model of ulcerative colitis which in turn provides recommendations and significance of choosing the best probiotic in our diet to prevent inflammation in UC patients and benefit from the homeostatic role of BI.

## Figures and Tables

**Table 1:** Criteria for scoring macroscopic colonic damage.

Score	Criteria
0	No damage
1	Hyperemia, no ulcers
2	Linear ulcer with no significant inflammation
3	Linear ulcer with inflammation at one site
4	Two or more sites of ulceration/inflammation
5	Two or more major sites of ulceration and inflammation or one site of ulceration/inflammation, extending >1 cm along the length of the colon
6–10	If damage covers >2 cm along the length of the colon, the score is increased by one, for each additional centimeter of involvement

A lower score represents a healthier rat (modified from Peran et al., 2007 [11]).

**Table 2:** Microscopic colonic damage scores.

	Group	*N*	Mean	Std. error mean	*t* value	*p* value
Colonic damage scores	Control	49	3.1837	.14775	10.36	<0.001
	BI	44	.9773	.14353		

Statistically significant difference between groups; higher damage was seen in control group.

**Table 3:** Comparison of histological scores between control and BI groups.

Group statistics-independent samples *t*-test								
	Group	*N*	Mean	Std. deviation	Std. error mean	*t* value	df	^*∗*^Sig. (2-tailed)
								*p* value
Mucous Architecture (0, normal; 3, maximal damage)	Control	49	1.8367	.62406	.08915	9.646	91	0.000
	BI	44	.6818	.51817	.07812			
Degree of cellular infiltration (0, normal; 3, maximum infiltration)	Control	49	1.8776	.48445	.06921	11.921	91	0.000
	BI	44	.7045	.46152	.06958			
Extent of muscle thickening (0, normal; 3, maximal thickness)	Control	49	.7959	.57661	.08237	2.526	91	0.013
	BI	44	.5000	.54984	.08289			
Crypt abscesses (0, absent; 1, present)	Control	49	.9388	.24223	.03460	21.602	91	0.000
	BI	44	.0227	.15076	.02273			
Goblet cell mucous depletion (0, absent; 1, present)	Control	49	1.0000	.00000	.00000	19.339	91	0.000
	BI	44	.1136	.32104	.04840			
Total score	Control	49	6.4490	1.00127	.14304	19.464	91	0.000
	BI	44	2.0227	1.19083	.17952			

^*∗*^Difference between control and BI groups was significant on all parameters under study. Control group had higher damage compared to BI group (*p* < 0.01).
